# Impact of xerostomia on the quality of life of patients submitted to head and neck radiotherapy

**DOI:** 10.4317/medoral.23131

**Published:** 2019-10-27

**Authors:** Michele Lopes do Nascimento, Andreza Barkokebas Santos de Farias, Alessandra Tavares Carvalho, Raylane Farias de Albuquerque, Lucas Nascimento Ribeiro, Jair Carneiro Leão, Igor Henrique Morais Silva

**Affiliations:** 1 Residency in Dentistry Oncology, Hospital do Cancer de Pernambuco - Recife, Pernambuco, Brazil; 2 Executive Secretary of Health Regulation / State Health Secretariat of Pernambuco. - Recife, Pernambuco, Brazil; 3 Professor of Oral Medicine, Oral Medicine unit, Departamento de Clinica e Odontologia Preventiva, Universidade Federal de Pernambuco – Recife, Pernambuco, Brazil; 4 Residency in Dentistry Oncology, Hospital do Cancer de Pernambuco - Recife, Pernambuco, Brazil.; 5 Coordinator of Residency in Dentistry Oncology, Hospital do Cancer de Pernambuco - Recife, Pernambuco, Brazil

## Abstract

**Background:**

The aim of the present work was to evaluate the impact of xerostomia on the quality of life of patients who underwent radiotherapy in the head and neck region.

**Material and Methods:**

This was a cross-sectional, quantitative study. The sample comprised 40 patients whose xerostomia was classified through the xerostomia inventory and the quality of life evaluated through the oral health impact profile questionnaire (OHIP).

**Results:**

The majority of participants were male (75%), mean age 58.7 years. According to the degree of severity of the xerostomia, the average score among the participants was 36 points, this being considered moderate xerostomia. A significant impact was observed, with the median score 11 points, with the highest scores in the domains related to functional limitation, physical pain and physical disability. The majority of the participants (97.5%) had reduced salivary flow after the end of radiotherapy. There was a significant positive correlation between the degree of xerostomia and reduced quality of life, Pearson correlation 0.5421, (*p*< 0.05).

**Conclusions:**

Based upon the results it is concluded that xerostomia has a negative impact on the quality of life of patients who undergo radiotherapy in the head and neck region.

** Key words:**Head and neck neoplasms, radiotherapy, xerostomia, quality of life.

## Introduction

Radiotherapy (RT) and surgery are described as standard therapies for early and locally advanced malignant tumors in the head and neck region. For advanced tumors, concomitant radiochemotherapy has also been used (1). Despite being one of the most used treatments, radiotherapy still produces important acute and long-term side effects for the oral cavity (2). Radiotherapy in the head and neck region is typically associated with toxicities that can have profound effects on the patient's quality of life. Among the most common are mucositis, xerostomia, dysgeusia, dysphagia, trismus, dermatitis and candidiasis (3).

Xerostomia is the most common oral complication of RT when the irradiated area involves the oral and maxillofacial complex, which may occur both during or after radiotherapy (4,5). Radiation-induced xerostomia depends on the cumulative doses of radiation on the head and neck region, in the first week of conventional RT, salivary flow decreases from 50 to 60%, after 7 weeks it diminishes to approximately 20% and continues to decline for up to several months after RT (2).

Damage to the salivary glands has generally shown a reduction in salivary flow, which can translate into a subjective sensation dry mouth (xerostomia), taste disturbance, difficulty speaking, swallowing, chewing and increased risk of caries, pain and burning of the mouth, all of which culminates in negative consequences on the quality of life (4,6,7). A variety of methods are currently available for the evaluation of radiation-induced xerostomia, however, dry mouth is a subjective experience and its assessment should rely on patient self-reports (8).

Over the last decade, there has been an increase in the use of quality of life measures in clinical trials. Many instruments were designed to measure the impact of oral health on quality of life. Slade and Spencer introduced the Oral Health Impact Profile (OHIP-49), a questionnaire containing 49 questions that capture seven conceptually formulated dimensions (functional limitation, physical pain, psychological discomfort, physical disability, social disability, and incapacity) (9). In 1997, Slade published a reduced questionnaire with the same dimensions (OHIP-14) that confirmed results comparable to those obtained with the original OHIP-49 (10). The OHIP-14, the abbreviated form of OHIP-49, is reported as a useful instrument for use in clinical environment with good reliability, validity and precision (10,11).

The evaluation of the effect of oral diseases and social conditions may be of great value to researchers, and studies on quality of life may guide practitioners to most effective treatments in patients with cancer (12). In view of the above, it is observed that studies that evaluate xerostomia and its consequences are of considerable importance, since it is a complex condition may have negative effects on the quality of life of individuals who need to undergo radiotherapy in the head and neck region. Thus, the present study aimed to evaluate the impact of xerostomia on the quality of life of patients who underwent radiotherapy in the head and neck region.

## Material and Methods

The study was an analytical cross-sectional with quantitative approach whose sample comprised 40 patients treated at a referral hospital in the state of Pernambuco (Brazil), from July to November of 2016. All patients were submitted to radiotherapy in the head and neck region and complained of xerostomia when first seen at the dental clinic of the hospital. Among the inclusion criteria of the study were the minimum age of 18 years and diagnosis of malignant neoplasia in the head and neck region. In addition, patients should have been treated with two-dimensional radiotherapy alone or concomitantly with chemotherapy or adjuvant surgery, the total dose should be equal to or greater than 50Gy and the treatment should include the larger salivary glands, oral cavity or oropharynx. Patients with other possible causal factors of xerostomia, such as those with diabetes mellitus, autoimmune, infectious and collagen diseases, and patients who used drugs that could interfere with salivary flow (antidepressants, benzodiazepines, anti- hypertensives, among others). Patients with indication for palliative radiotherapy or who were unable to answer the questions were also excluded.

Xerostomia was evaluated using the translated and adapted version of the Xerostomia Inventory (XI), validated by Mata *et al*., originally developed by Thomson *et al*. (13,14). This is a questionnaire composed of 11 questions that contemplates the symptomatological and behavioral aspects of xerostomia. For each of the 11 items, there are five response options, which indicates how often each of the reported symptoms has occurred in the last four weeks. Each answer has a value: the answer 'Never' is 1, 'Almost never' is 2, 'Occasionally' is 3, 'Relatively often' is 4, and 'Frequently' is 5. At the end, the scores are summed and generate a single value between 11 and 55, 11 being classified as mild xerostomia and 55 severe xerostomia.

Sialometry with determination of unstimulated salivary flow (USF) was also performed using the technique described by Sreebny and Vissink (15). Values above 0.25 ml/min of USF were considered normal. All saliva collected in five minutes was conditioned in a heavy disposable container before and after the start of collection. For the calculation of total salivary flow, assuming that 1g of saliva corresponds to 1 ml, the following conversion formula was used (15,16).

Salivary flow (ml/ min) = [Weight of tube after (g) – Weight of tube before (g)] / Time of saliva collection (min).

After the xerostomia was measured and sialometry was performed, a clinical examination of the oral cavity was carried out, in order to evaluate the presence of mucosal lesions, osteoradionecrosis and caries on the cervical, incisal or root regions. For the data collection, a questionnaire was initially used with questions addressing socio-demographic aspects in order to present the patient profile, and for the analysis of the quality of life, the summarized version of the Oral Health Impact Profile (OHIP 14) was used, translated and adapted into portuguese, proposed by Slade in 1997 and validated by Oliveira and Nadanovsky in 2005 (10,12).

The OHIP-14 instrument consists of seven dimensions: Functional Limitation, Physical Pain, Psychological Discomfort, Physical Inability, Psychological Inability, Social Inability and Incapacity. There are five answer options with a code for the 14 questions: 'Never' is worth 0 point, 'Rarely' 1 point, 'Sometimes' 2, 'Repeatedly' 3, and 'Always' is worth 4. In the OHIP-14 the scale ranges from 0 to 56 points, and the higher the score, the worse the quality of life is (10).

For statistical analysis, the data were described with frequency distributions for the categorical variables and with medians and respective standard deviation when the quantitative variable presented normal distribution, otherwise the mean and interquartile range were presented. In the analysis of the association of the time of therapy and explanatory variables the Fischer Chi-Square test were applied in the comparison of categorical variables and for the quantitative variables the ANOVA test was applied, with Bonferroni post-test for the comparison of means, and Kruskall-Wallis test for the comparison of medians. A Pearson correlation measure was estimated and the hypothesis in the analysis of the relation between the degree of xerostomia and quality of life of the patients was tested. The statistical significance adopted in the study was 5% (*p*<0.05) and the software used in the analysis was STATA version 12.0.

This study was approved by the Ethics and Research Committee of the Pernambuco Society against Cancer, with certification number 1.602.883.

## Results

The study included 40 patients, most of whom were male (75%), with a mean of 58.7 years of age. Stratified in age groups, 57.5% of patients were between 50 and 64 years of age. Regarding the origin of the patients, 42.5% were residing in the countryside of the state of Pernambuco, Most of the patients were retired and 17.5% had agriculture as occupation (Table 1).

Regarding the clinical aspects of the patients (Table 2), the most common type of neoplasm was oral squamous cell carcinoma OSCC (82.5%), 40% of the tumors were located in the oropharynx and 32.5% in the oral cavity. Alterations in oral cavity were observed in half of the patients, caries of radiation being the most frequent alteration (60%). Out of the 40 patients studied, 28 (70%) had chemotherapy associated with radiotherapy.

**Table 1:**
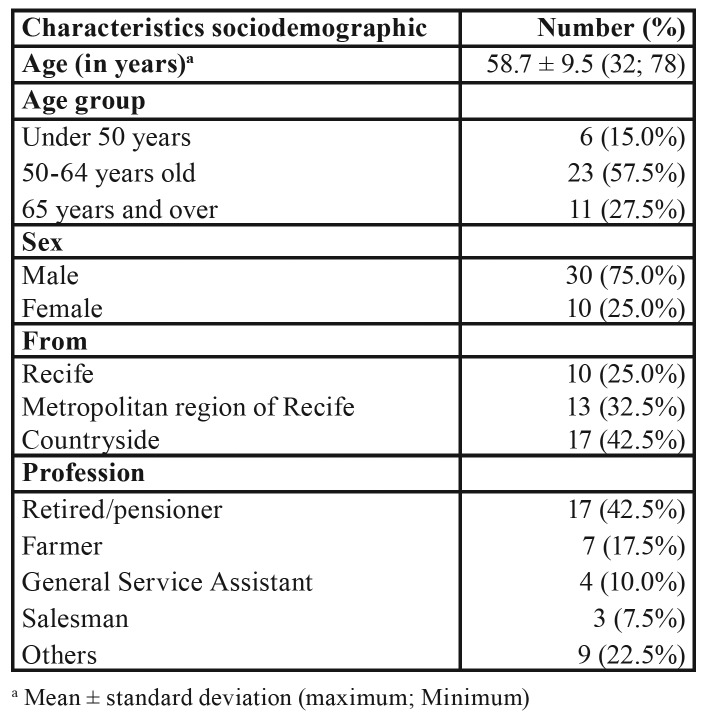
Sociodemographic profile of patients with diagnosis of malignant lesions in the head and neck region treated at Hospital de Cancer de Pernambuco.

**Figure 1:**
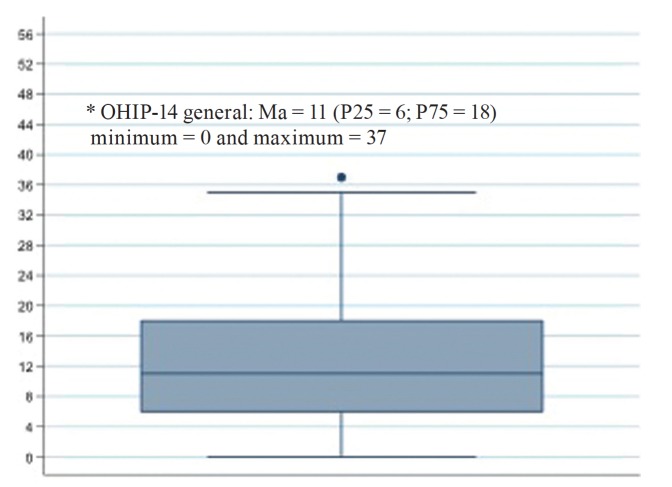
Overall mean score of the oral health impact profile (OHIP-14) of patients diagnosed with malignant lesions in the head and neck region treated at the Hospital de Cancer de Pernambuco.

Regarding the time of conclusion of radiotherapy, most participants (45%) had between 7 and 18 months. Regarding the degree of severity of the xerostomia, the average score among the participants was 36 points. It was observed that 39 (97.5%) of the patients had a decrease in salivary flow, whereas only one patient had a normal salivary flow, that is, a flow above to 0.25 ml/min (Table 2). Regarding the quality of life (Fig. 1), the median OHIP-14 score of the respondents was 11 points, with a minimum score of zero and a maximum of 37 points, with the majority of participants having a score above the median, demonstrating a significant negative impact on the quality of life of these patients.

**Table 2:**
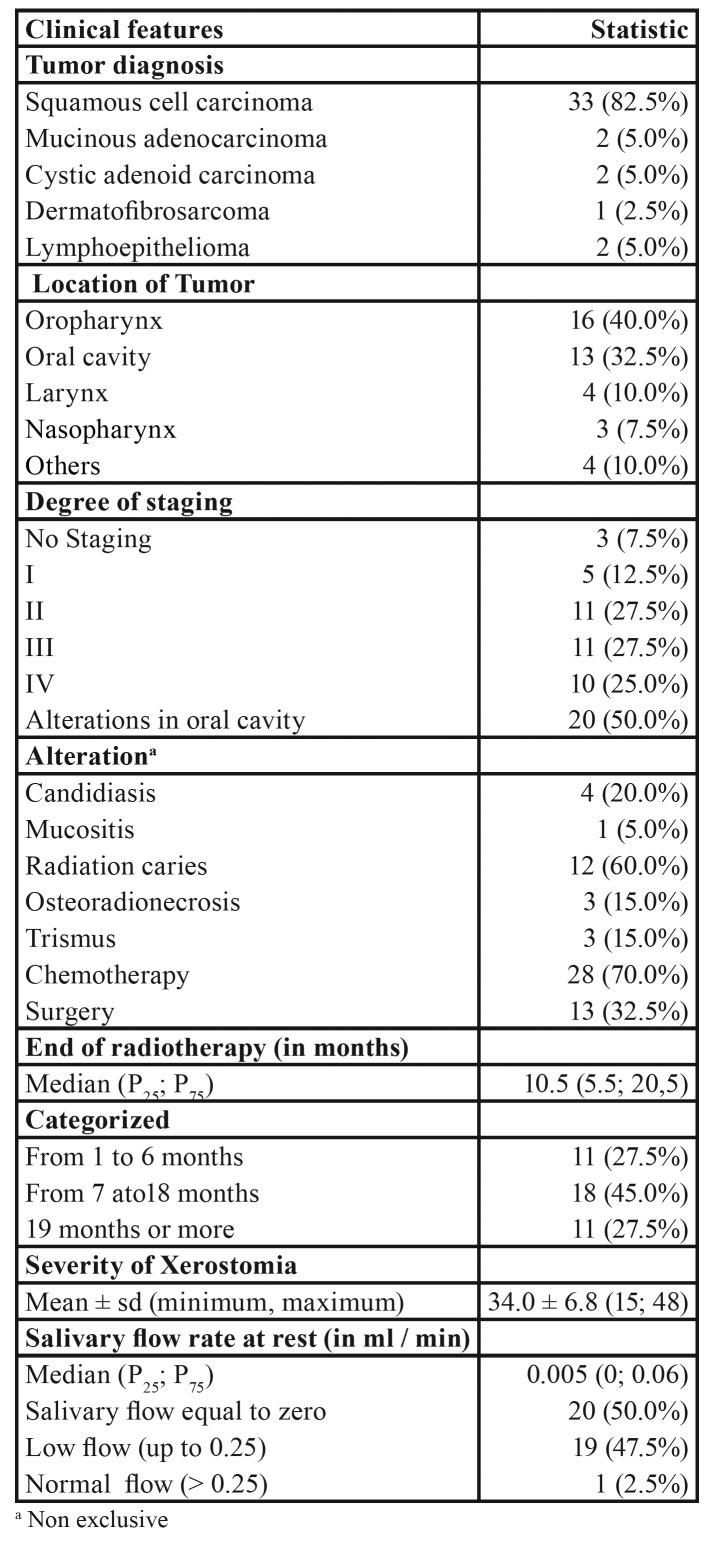
Clinical aspects of patients diagnosed with malignant lesions in the head and neck region treated at Hospital de Cancer de Pernambuco.

**Figure 2:**
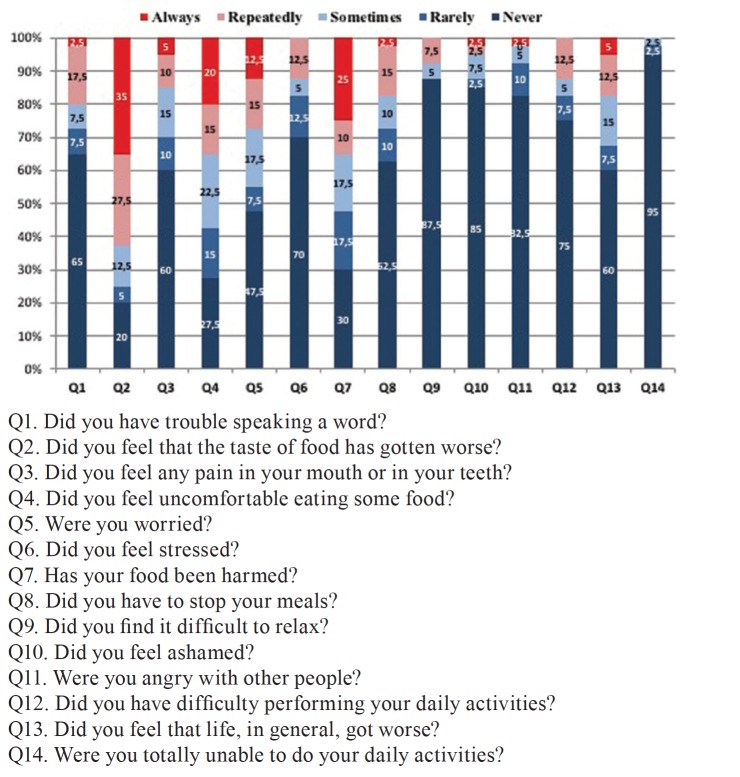
Percentage distribution of the quality of life related issues of the oral health impact profile (OHIP-14) of patients diagnosed with malignant lesions in the head and neck region treated at Hospital de Câncer de Pernambuco.

**Figure 3:**
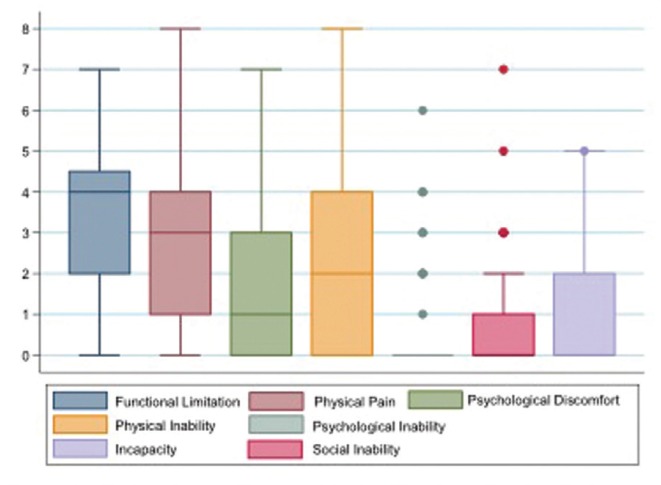
Mean score of the oral health impact profile (OHIP-14) by domain of patients diagnosed with malignant lesions in the head and neck region treated at the Hospital de Cancer de Pernambuco.

Analyzing the scores on the quality of life scale through the oral health impact profile (Fig. 2), we observed that questions 2 ("Did you feel that the taste of food has gotten worse?"), 4 ("Did you feel uncomforTableeating any food? ") and 7 ("Has your food intake gotten impaired?") were the ones with a higher frequency of answers “always” or “repeatedly” between those surveyed. However the questions 9 ("Did you find it difficult to relax?"), 10 ("Did you feel embarrassed?") and 14 ("Have you become totally unable to do your daily activities?") were the ones with a lower frequency of answers “always” or “repeatedly”. This reflects in the analysis by domains where the highest scores, and consequently a worse quality of life, are in the domains related to functional limitation, physical pain and physical disability (Fig. 3).

Regarding the influence of xerostomia on patients' quality of life, it was observed that there was a positive correlation when Pearson's correlation was performed (r = 0.5421; *p*<0.05), thus demonstrating that there was an influence of xerostomia in the decrease of quality of life of the patients studied. Stratified by the time of conclusion of radiotherapy, this correlation is statistically significant in patients with more than 6 months of radiotherapy, with values ​​equal to 0.6680 (*p*<0.05) and 0.6031 (*p*<0.05) for patients with time of conclusion between 7 and 18 months and over 18 months, respectively.

## Discussion

Xerostomia is the most common oral complication in patients who undergo radiotherapy of the head and neck and may appear during or after radiotherapy (4,17). The duration and intensity of these effects are determined by factors such as dose per fraction, total dose of radiation, volume of irradiated gland, dose distribution in tissue volume and association with chemotherapy (18). Corroborating with the literature, in this study more than half of the patients who presented with xerostomia received radiation in the oral cavity and oropharynx region, covering the major salivary glands.

Several authors affirm that hypofunction of the salivary gland caused by radiotherapy in the head and neck is a strongly associated factor in the etiology of xerostomia (17,19,20). Tiwana *et al*. in a study of patients with head and neck cancer after conventional radiotherapy observed that there was a decline in the salivary flow of patients during radiotherapy and that after 6 months of treatment, 39% of the patients had a stimulated salivary flow smaller than 0.01 ml/min. (19). In this study, it was observed that of the 40 patients who reported xerostomia after radiotherapy, practically all of them had reduced salivary flow and half of the sample presented salivary flow equal to zero. This demonstrates that hyposalivation is an important factor associated with the sensation of buccal dryness described by patients receiving radiation therapy to the head and neck.

The literature shows that the reduction of salivary secretion can translate beyond xerostomia into oral discomfort, altered taste, difficulties in speech, chewing, swallowing and increased risk of dental caries and secondary infections (7). Our data are in agreement with the literature, when a good part of the sample presented candidiasis and more than half developed caries of radiation. Carvalho *et al*. show that salivary flow alterations induced by radiotherapy of the head and neck are the main responsible for a cariogenic environment, and caries of radiation can occur even in teeth not exposed to radiation (21).

The present study aimed to evaluate xerostomia through three parameters, the first one related to subjective feeling through the Xerostomia Inventory (XI), the second relating xerostomia to quality of life through the OHIP-14 questionnaire and the third, using clinical evidence of hypofunction of the salivary gland, using sialometry as an objective measure. According to Sasportas *et al*. these three parameters are important in the evaluation of a patient with xerostomia, because despite several methods described in the literature to standardize a classification for xerostomia, clinical evaluation often underestimates subjective severity, besides the fact that xerostomia is a condition which negatively affects the patient's quality of life (4).

A study conducted in Canada, evaluating the quality of life related to oral health in patients with dry mouth, found that xerostomia had an important influence on well-being and quality of life (22). The present study demonstrated that there was influence between xerostomia and patients' quality of life, with a positive correlation when Pearson's correlation was performed. It is observed that the higher the degree of severity of the xerostomia, the worse the quality of life, corroborating with findings in the literature (4,6).

In this study, there was a statistically significant association with the time of conclusion of radiotherapy in the categorized time analysis, with a greater impact of xerostomia after 6 months and 18 months. Radiation-induced xerostomia is the most prevalent and prominent long-term side effect in patients after radiation in the head and neck region, and it is usually related to a decrease in the patient’s quality of life (23).

Analyzing the distribution of the questions related to the quality of life of the oral health impact profile (OHIP-14) of the patients investigated, we observed that questions 2 ("Did you feel that the taste of food has gotten worse?"), 4 ("Did you feel uncomforTableeating any food? ") and 7 ("Has your food intake gotten impaired?") were those with a higher frequency of answers “always” or “repeatedly” among the patients. This reflects in the analysis by domains where the highest scores, and consequently a worse quality of life, are in the domains related to functional limitation, physical pain and physical disability. In a study by Niklander *et al*., observing risk factors, hyposalivation and oral health impact on quality of life, found that patients with xerostomia obtained higher scores in all OHIP-14 domains comparing to control patients, with greater impact on the domains related to psychological discomfort, psychological incapacity and physical pain (24).

As already mentioned, the results of this study demonstrated that xerostomia in patients who underwent radiotherapy in the head and neck region has an important effect on quality of life. It is relevant to say that the treatments available for xerostomia are generally not effective; sialogogues have side effects and are not always efficient. The effects of saliva substitutes are limited and the level of patient satisfaction is generally low (6,25). Among the therapies that have been highlighting is the use of low power laser, for promoting biomodulation of cellular metabolism, analgesia and anti-inflammatory effects, without mutagenic and photothermal effects (18). Therefore, the prevention of radiation-induced xerostomia can have a significant impact on long-term quality of life. Intensity-modulated radiotherapy (IMRT) is described as a way of preventing or minimizing the degree of xerostomia, widely used as the most effective solution in terms of benefits (4,26). Sanguineti *et al*., have provided evidence that patients whose parotids have a significantly reduced radiation dose during IMRT for oropharyngeal cancer will be less likely to be dependent on artificial saliva at 1 and 2 years post-treatment (27). However, this type of radiotherapy treatment is not yet a reality in all public network oncology services, in fact no patient in this study was treated with IMRT.

Conclusion

Based upon the results of the present study it is concluded that xerostomia has a negative impact on the quality of life of patients who undergo radiotherapy in the head and neck region.
